# Comparative Analysis of CTRP-Mediated Effects on Cardiomyocyte Glucose Metabolism: Cross Talk between AMPK and Akt Signaling Pathway

**DOI:** 10.3390/cells10040905

**Published:** 2021-04-14

**Authors:** Ling Li, Muhammad Aslam, Benedikt H. Siegler, Bernd Niemann, Susanne Rohrbach

**Affiliations:** 1 Institute of Physiology, Justus Liebig University Giessen, 35392 Giessen, Germany; Benedikt.H.Siegler@chiru.med.uni-giessen.de (B.H.S.); Susanne.Rohrbach@physiologie.med.uni-giessen.de (S.R.); 2 Experimental Cardiology, Department of Cardiology and Angiology, Justus Liebig University Giessen, 35392 Giessen, Germany; muhammad.aslam@physiologie.med.uni-giessen.de; 3 Department of Cardiac and Vascular Surgery, Justus Liebig University Giessen, 35392 Giessen, Germany; bernd.niemann@chiru.med.uni-giessen.de

**Keywords:** adiponectin, glucose metabolism, AMPK, C1q family, Akt, AdipoR

## Abstract

C1q/tumor necrosis factor -alpha-related proteins (CTRPs) have been shown to mediate protective cardiovascular effects, but no data exists on their effects on glucose and fatty acid (FA) metabolism in cardiomyocytes. In the present study, adult rat cardiomyocytes and H9C2 cardiomyoblasts were stimulated with various recombinant CTRPs. Glucose or FA uptake, expression of genes involved in glucose or FA metabolism and the role of the AMP-activated protein kinase (AMPK) and Akt were investigated. Although most CTRPs induced an increase in phosphorylation of AMPK and Akt in cardiomyocytes, mainly CTRP2, 7, 9 and 13 induced GLUT1 and GLUT4 translocation and glucose uptake in cardiomyocytes, despite high structural similarities among CTRPs. AMPK inhibition reduced the CTRPs-mediated activation of Akt, while Akt inhibition did not impair AMPK activation. In addition, CTRP2, 7, 9 and 13 mediated strong effects on the expression of enzymes involved in glucose or FA metabolism. Loss of adiponectin receptor 1, which has been suggested to be involved in CTRP-induced signal transduction, abolished the effects of some but not all CTRPs on glucose metabolism. Targeting the AMPK signaling pathway via CTRPs may offer a therapeutic principle to restore glucose homeostasis by acting on glucose uptake independent of the Akt pathway.

## 1. Introduction

Adipose tissue is not only the major storage depot for triglycerides but also an endocrine organ that secretes so-called adipokines into circulation. Unlike other adipokines, adiponectin (acrp30) is decreased in obesity and directly sensitizes the body to insulin [1]. Adiponectin plays important roles in the regulation of insulin sensitivity, atherosclerosis and cardiometabolic disease [1]. Adiponectin has direct beneficial effects on cardiomyocytes in several pathological heart conditions, including cardiac hypertrophy, but it also preserves cardiomyocyte function by exerting beneficial effects on glucose and lipid metabolism [2,3]. It also stimulates basal as well as insulin-stimulated glucose uptake and Akt phosphorylation in cardiomyocytes through an AMP-activated protein kinase (AMPK)- and adiponectin receptor-1 and -2 (AdipoR1/2)-dependent mechanism [4].

Some years ago, a family of structural and functional adiponectin paralogs, comprising 15 members so far, was discovered and designated as C1q/tumor necrosis factor-alpha-related proteins (CTRPs) [5]. CTRPs and adiponectin share a common structure consisting of a signal peptide at the N terminus, a short variable region, a collagenous domain, and a C-terminal globular domain that is homologous to complement component 1q [6]. Among these, CTRP9 shares the highest degree of amino acid identity (51%) with the globular domain of adiponectin [7]. In addition, CTRP2 (42%), CTRP5 (41%), CTRP7 (43%) and CTRP13 (39%) also own high amino acid similarity to adiponectin at the globular domain, suggesting that those CTRPs might have similar biologic properties to adiponectin [7,8]. CTRPs are predicted to be secreted proteins that circulate in the serum and form heteromultimers with adiponectin or other CTRPs in vivo. Circulating levels of CTRP1, CTRP3, CTRP9, CTRP12 and CTRP15 were reduced in obese mice [9]; CTRP7 and CTRP9 were shown to be increased in patients with insulin resistance, type 2 diabetes mellitus or metabolic syndrome [10,11], while other CTRPs were reduced in such patients [12].

Unlike adiponectin, the CTRPs are widely expressed outside adipose tissue and may exert their biological effects in a paracrine or autocrine fashion [5]. CTRP1, CTRP5, CTRP12 and CTRP13 have been reported to stimulate glucose uptake in adipocytes, hepatocytes or myotubes, while CTRP1, CTRP3, CTRP5, CTRP9 and CTRP15 promote uptake and oxidation of fatty acids (FAs) [9]. Loss or gain of function studies in mice suggested a significant but contrasting effect of CTRPs on metabolism. While deletion of CTRP7 improved glucose metabolism in mice [10], loss of CTRP1 and CTRP9 resulted in impaired glucose metabolism [13,14]. Accordingly, mice with overexpression of CTRP9 or CTRP2 were protected from insulin resistance and diet-induced obesity [15,16]. Unlike these, loss of CTRP3 had minimal or no impact on whole body glucose metabolism or insulin sensitivity [17]. In vitro experiments in different cell types also showed that CTRP3 reduced FA synthesis and neutral lipid accumulation [18], while CTRP15 promoted FA uptake, in part by upregulating the expression of genes involved in lipid uptake (CD36, FATP1, Fabp1 and Fabp4) [19]. Some, but not all, of the observed effects appear to be mediated via enhanced insulin signaling [9]. Other signaling pathways that have been described to be activated by CTRPs include p44/42 MAPK, Akt, AMPK, p38MAPK, STAT3 and ERK5 [6,9,20,21]. So far, the most relevant CTRPs, in terms of cardioprotective effects, appear to be CTRP1, CTRP3, CTRP9 and CTRP15 [9,22,23,24,25,26]. CTRP1 protects against myocardial ischemic injury by reducing apoptosis and inflammatory response. CTRP3 improves survival and restores cardiac function after myocardial infarction and attenuates postischemic pathological remodeling, which involves Akt–HIF1α–VEGF signaling. CTRP9 reduces myocardial infarct size and cardiomyocyte apoptosis following ischemia-reperfusion, improves cardiac function in diabetic mice and attenuates adverse cardiac remodeling after myocardial infarction. CTRP15 ameliorates acute myocardial ischemic injury and exerts an antifibrotic effect on pressure overload-induced cardiac remodeling. Antihypertrophic effects have been attributed to CTRP1, CTRP3 and CTRP9.

Adiponectin mediates its metabolic effects mostly via its receptors, AdipoR1 and AdipoR2 [27]. In view of the structural similarities, these receptors may also mediate the CTRP effects. Indeed, AdipoR1 was identified as a putative receptor for CTRP9 [28,29]. However, others have suggested that CTRP receptors may be distinct from adiponectin receptors, since overexpression of the adiponectin co-receptor T-cadherin in multiple cell types does not result in enhanced binding of CTRP9 [7], and phosphorylation of AMPK induced by CTRP5 is unaffected by AdipoR1 or AdipoR2 depletion [30].

The overall aim of our study was to investigate the effects of CTRPs on cardiomyocyte metabolism. Therefore, we compared the effects of the adiponectin paralogs CTRP 1-15 on (1) glucose metabolism, (2) FA metabolism and (3) signaling pathways involved in the observed effects.

## 2. Materials and Methods

### 2.1. Generation and Purification of Recombinant CTRPs in Escherichia coli (E. coli)

Recombinant protein was produced by cloning full-length mouse CTRP1, 2, 3, 7, 9, 12, 13 CDS into pENTR™/D-TOPO^®^ (ThermoFisher Scientific, Darmstadt, Germany) and CTRP6, 10, 15 into pGEM^®^-T Easy (Promega Corporation, Walldorf, Germany), maintained in the *E. coli.* strain TOP10. Using the Gateway^®^ Technology, CTRP1, 2, 3, 7, 9, 12, 13 were transferred into pDEST17. CTRP6, 10, 15 were cloned into pMAL-c2X following restriction digestion and ligation. Afterward, all vectors were sequenced (Eurofins Genomics, Ebersberg, Germany) and maintained in the *E. coli* strain BL21-AI (ThermoFisher Scientific). CTRPs with an N-terminal histidine (His)-tag (pDEST17) were isolated from the lysed bacterial pellet using nickel-affinity columns (Amocol Bioprocedures Ltd, Teltow, Germany) and eluted with imidazole containing buffer. CTRP fusion proteins (pMAL-c2X) with an N-terminal maltose binding protein (MBP) were purified with amylose resin (New England Biolabs Inc., Ipswich, MA, USA) and eluted with maltose. All fusion proteins were dialyzed against phosphate-buffered saline (PBS). Potential endotoxin contaminants were removed with the EndoTrap^®^ red endotoxin removal kit (Hyglos GmbH, Bernried, Germany). Absence of endotoxin was verified with the Pierce Chromogenic Endotoxin Quant Kit (ThermoFisher Scientific), where 1 endotoxin unit (EU) equals approximately 0.1 to 0.2 ng endotoxin. The lower limit of the kit is 0.01 EU/mL (Supplementary Figure S2). A concentration response of all CTRPs was established and was used at optimal concentration of 4 µg/mL in all experiments. An unrelated His-tagged or MBP-tagged protein was utilized at the same concentration and served as control. Adenine 9-β-D-arabinofuranoside (AraA, Jena Biosciences, Jena, Germany) or the inhibitor VIII (inhibitor. VIII, Enzo life sciences, Lause, Switzerland) was used for 30 min to inhibit AMPK or Akt1/2 phosphorylation.

### 2.2. H9C2 Cardiomyoblast Culture and Transfection

The H9C2 cardiomyoblast cell line was obtained from the American Type Culture Collection (Manassas, VA, USA). H9C2 cardiomyoblasts were maintained in high glucose Dulbecco’s Modified Eagle Medium (DMEM) supplemented with 10% fetal calf serum (FCS) and 1% penicillin/streptomycin under an atmosphere of 5% CO_2_ at 37 °C. The cells were treated in serum-free medium with CTRPs (4 µg/mL) as indicated. Twenty-four hours before transfection with pQBI25 HA-GLUT-4, cells were trypsinized and transferred to 6-well plates (2 × 10^5^ cells/well). Transfection was performed with jetPRIME ™ transfection reagent (Polyplus Transfection, Illkirch, France) following the manufacturer instructions. Twenty-four hours before transfection with siRNA, cells were trypsinized and transferred to 6-well plates (5 × 10^5^ cells/well). AdipoR1, AdipoR2 and control siRNA (FlexiTube siRNA, Qiagen, Hilden, Germany) oligonucleotides were transfected to the cells at a concentration of 0.5 nmol/L with Lipofectamine^®^ RNAiMAX (ThermoFisher Scientific). The sequences had been digitally searched, and no similarity to other genes was found in current databases. The control cells were transfected with control siRNA oligonucleotides, with no known target in mammalian genomes. Forty-eight hours after siRNA transfection and after serum starvation for 24 h, cells were treated, as indicated.

### 2.3. Isolation of Adult Rat Cardiomyocytes, Endothelial Cells and Fibroblasts

Ventricular heart muscle cells were isolated from rats, as described previously [31]. Briefly, hearts were excised under deep anesthesia, transferred rapidly to ice-cold saline, and mounted on the cannula of a Langendorff perfusion system. Hearts were perfused first for 5 min in a non-recirculating manner with a calcium-free perfusion buffer, then for 20–25 min in a recirculating manner in a buffer supplemented with collagenase and 25 µmol/L calcium. Thereafter, ventricular tissue was minced and incubated for another 5 min in recirculating buffer. The remaining cell solution was filtered through a 200 µm nylon mesh. The suspension was centrifuged at 400 rpm for 3 min to pellet down the cardiomyocytes and the supernatant contained mostly the endothelial cells (ECs) and fibroblasts (FBs). Cardiomyocytes were resuspended in buffer with a stepwise increase in calcium and, finally, transferred to culture medium (M199 supplemented with 2 mM carnitine, 5 mM creatine and 5 mM taurine). Rat cardiomyocytes were attached to culture dishes by precoating the dishes with 4% FCS. The remaining cells (supernatant after centrifugation) were centrifuged at 250× *g* for 10 min, and the pellet was resuspended in 1 mL of endothelial cell basal medium (PromoCell, Heidelberg, Germany) and incubated with magnetic beads (ThermoFisher Scientific) precoated with antirat CD31 (ThermoFisher Scientific) for 1 h at 4 °C with end-to-end rotation. The microvascular endothelial cells coupled to magnetic beads were separated with a magnet, washed with endothelial cell basal medium and seeded on 35 mm culture dishes. This procedure removed over 95% of endothelial cells from the mixture, and the rest of the cells were seeded as cardiac fibroblasts in M199 medium supplemented with 10% FCS. All animals were handled in accordance with the directive 2010/63/EU of the European Parliament on the protection of animals used for scientific purposes.

### 2.4. Western Blotting

Cells were homogenized in a buffer containing 50 mM Tris HCl, 150 mM NaCl, 5 mM Ethylenediaminetetraacetic acid (EDTA), 0.1% Sodium dodecyl sulfate (SDS), 1% sodiumdeoxycholate and protease and phosphatase inhibitor cocktails (Sigma, Schnelldorf, Germany). After sonication and centrifugation, protein concentration was measured with the Pierce™ BCA Protein Assay Kit (ThermoFisher Scientific). A total of 20 µg of protein were loaded on an SDS-PAGE gel and transferred to a nitrocellulose membrane. Following blocking, filters were incubated with antibodies directed against His, MBP, phosphor–AMPK (Thr172), alpha–AMPK, phospho–AS160 (Thr642), phospho–Akt (Thr308), Akt, ACC, phospho–ACC (all Cell Signaling Technology, Europe B.V., Frankfurt am Main, Germany), GLUT1, GLUT4 (both from Cushman, S.W. [32]), MBP (NEB), AS160 (Merck Millipore, Darmstadt, Germany) and GAPDH (Abcam, Cambridge, UK). After incubation with peroxidase-conjugated secondary antibody, blots were subjected to the enhanced chemiluminescent detection method with the Fusion FX7 imaging system (Peqlab, Erlangen, Germany).

### 2.5. RNA Isolation, RT-PCR and qPCR

Total RNA from different rat organs and from cardiomyocytes or H9C2 cells was isolated using TriFast (Peqlab, Erlangen, Germany), according to the manufacturer’s instructions. Prior to cDNA synthesis, integrity and quality of the RNA was confirmed by gel electrophoresis, and the concentration was determined by measuring UV-absorption. Reverse transcription of RNA samples (500 ng total RNA) was carried out for 30 min at 42 °C using the SuperScript™ III First-Strand cDNA Synthesis Kit (ThermoFisher Scientific). RT-PCR was performed for 18S rRNA, GAPDH, acrp30, CTRP1-7, CTRP9, 10, 12, 13, 15, BNP, eNOS and fibronectin. Amplification products were subjected to electrophoresis through 1.5% agarose gels, stained with GelRed (VWR, Darmstadt, Germany) and visualized with the Fusion FX7 imaging system (Peqlab, Erlangen, Germany). PCR products were excised from the gel, purified and directly sequenced (Eurofins Genomics). Real-time PCR and data analysis for GAPDH, HPRT-1, 18S rRNA, GLUT1, GLUT4, hexokinase, PFK1, ACC1, CD36, FATP1, VLCAD, LCAD and MCAD were performed using the M × 3000P Multiplex Quantitative PCR System (Stratagene, San Diego, CA, USA), as described previously [29]. Each assay was performed in duplicate, and validation of the PCR runs was assessed by evaluation of the melting curve of the PCR products (primer sequences in Supplementary Table S1) and by the slope and error obtained with the standard curve. All data of mRNA are given as relative units of the average of three different house-keeping genes (18S rRNA, GAPDH, HPRT-1).

### 2.6. Measurement of Glucose Uptake

Cells were starved in DMEM without serum and glucose for 3 h, then stimulated with CTRPs in serum-free DMEM containing 0.05 µCi/mL 2-[1-^14^C]-2-deoxyglucose (DG) and 50 µM 2DG for 1 h. Cells were then washed three times with ice-cold PBS, treated with 500 mM NaOH and incubated overnight at 37 °C. Radioactivity was measured by liquid scintillation counting. Glucose uptake was expressed as the fold-increase over the basal level and calculated in relation to the protein concentration of the sample.

### 2.7. Measurement of Fatty Acid Uptake

The use of fluorescently labeled FAs to study uptake was adapted from adipocytes and has been used in cardiomyocytes before [33]. Adult cardiomyocytes were plated into 24-wells. For detecting FA uptake, 10 µM of the fluorescent long-chain FA analog BODIPY™ 500/510 C1, C12 (ThermoFisher Scientific) was loaded into isolated cardiomyocytes for 5 min, and the cells were then washed with 0.5% Bovine serum albumin (BSA) in PBS twice. Intracellular fluorescence was measured immediately, using a Fluostar Optima microplate fluorometer (BMG Labtech, Ortenberg Germany; excitation 488 nm, emission 515 nm, cut-off 495 nm). The cells were then lysed in a buffer containing 50 mM Tris HCl, 150 mM NaCl, 5 mM EDTA, 0.1% SDS, 1% sodiumdeoxycholate and protease and phosphatase inhibitor cocktails to measure protein concentration of the samples.

### 2.8. Confocal Microscopy

Cells were grown on cover slips and stimulated, as indicated. For detection of GLUT4 translocation, H9C2 cells were transfected with pQBI25 HA-GLUT4 (kindly provided by S. W. Cushman) [32] prior to stimulation. In intact cells, the antibody can bind to HA-tagged GLUT4 only when the transporter is located in the membrane, since the HA epitope is cloned in an exofacial loop of GLUT4 [32]. After fixation with 3.7% formaldehyde for 20 min, the samples were washed and blocked with 5% newborn calf serum (NCS) and 5% BSA in PBS and, afterward, incubated with antibodies (all 1:100) directed against the extracellular domain of GLUT1 and GLUT4 or HA at 4 °C overnight. After washing, the samples were incubated with Cy3-coupled secondary antibodies (1:1000), followed by nuclear staining with TO-PRO™-3 Iodide (ThermoFisher Scientific). The samples were embedded, and images were obtained and processed using a LSM 510 confocal laser scanning microscope (Carl Zeiss, Jena, Germany).

### 2.9. Measurement of Cellular ATP Content, Viability and ROS Production

H9C2 cells were seeded in black 96-wells at 5 × 10^3^ cells/well and treated with an unrelated His-tagged protein, CTRP7 or CTRP9 (4 µg/mL) for 24 h, unless otherwise stated. Cellular ATP content was measured by phosphorylating glycerol, resulting in a fluorometric product proportional to the amount of ATP, with the ATP fluorometric Assay Kit (Sigma) at 535 nm (excitation) and 587 nm (emission) on an Infinite^®^ M200 microplate reader (Tecan, Männedorf, Switzerland). In order to estimate cell viability, cells were loaded with the CellTiter-Blue^®^ Reagent (Promega, Walldorf, Germany), according to the manufacturer’s instructions, and incubated for another two hours. Afterward, fluorescence was recorded at 560 nm (excitation) and 590 nm (emission). For the measurement of cellular reactive oxygen species (ROS) production, cells were loaded with 10 µM 2′,7′ –dichlorofluorescin diacetate (DCFDA, Sigma) for 30 min, washed with PBS and, afterward, incubated for 24 h with CTRP7 or CTRP9. Fluorescence was recorded at 485 nm (excitation) and 535 nm (emission). All data are expressed in relation to the according controls.

### 2.10. Statistical Analysis

All data are presented as mean ± SEM. Statistical analyses were performed with SigmaStat 3.5 software (Systat Software, Inc, San Jose, CA, USA). Data were analyzed for normal distribution (Shapiro–Wilk test) and variance (Levene test) and, subsequently, analyzed using student t test or ANOVA with post hoc analysis (Tukey Test or Holm–Sidak Test). *p* values of <0.05 were considered statistically significant.

## 3. Results

### 3.1. CTRPs Exist Ubiquitously in Rat Organs and Cardiac Cells

**Figure 1:**
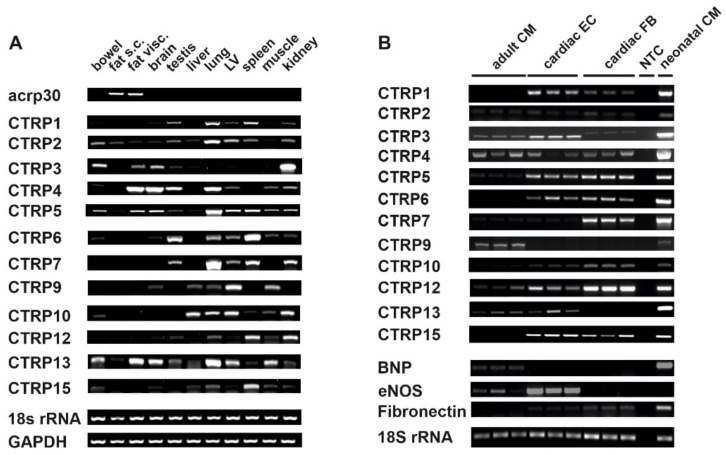
Analysis of the expressional pattern of the currently known C1q/tumor necrosis factor-alpha-related protein (CTRP) mRNAs by conventional RT-PCR in rats. CTRP expression in (**A**) various rat tissues and (**B**) different cardiac cells.

The PCR data showed (Figure 1A) that, unlike adiponectin that was mainly expressed in adipose tissues, CTRPs were widely expressed in various rat organs/tissues. The rat left ventricular (LV) tissue showed the expression of all CTRPs tested with highest expression of CTRP2, 5, 9, 10 and 13 and moderate expression of CTRP1, 4, 6 and 7. In order to identify the cell-specific expression of different CTRPs in LV, primary cells from adult rat hearts were isolated. As shown in Figure 1B, cardiomyocytes expressed CTRP2, 3, 4, 5, 7, 9, 12 and 13. Cardiac ECs, however, expressed all CTRPs tested except CTRP9. The cardiac fibroblast, on the other hand, expressed all the CTRPs except CTRP9 and 13. Purity of the isolated cardiac cells was demonstrated by the expression of cell-specific genes, such as brain natriuretic peptide (cardiomyocytes) or endothelial nitric oxide synthase (eNOS) for ECs.

### 3.2. Effect of CTRPs on AMPK and Akt-Dependent Glucose Uptake in Adult Cardiomyocytes

Adiponectin enhances FA oxidation and stimulates glucose uptake in cardiac muscle, and similar effects have also been proposed for some of the CTRPs in other cells types [9]. In order to investigate their functional effect on cardiomyocytes, full-length CTRPs were expressed in *E. coli*, purified by affinity chromatography (Supplementary Figures S1 and S2) and used in the experiments. GFP or MBP were expressed, accordingly, and served as treatment controls (Supplementary Figure S2A,B). Following its removal, all bacterial CTRP preparations were made endotoxin-free using a commercial endotoxin removal kit, as shown in methods and Supplementary Figure S2C.

**Figure 2:**
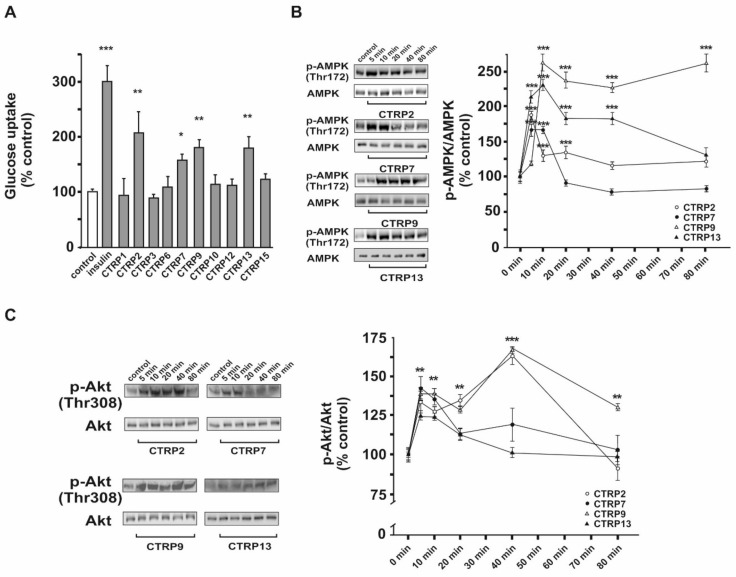
Glucose uptake and phosphorylation of AMPK and Akt in response to CTRPs in adult rat cardiomyocytes. (**A**) Following treatment with insulin (100 nM) or CTRPs for 1 h in DMEM without serum and glucose after 3 h glucose starving, glucose uptake was measured. (**B**) Western Blots were performed for phosphorylation of AMPK during the time course experiments for CTRP2, CTRP7, CTRP9 and CTRP13. Total AMPK served as loading control. The results of the densitometric evaluation are shown on the right. (**C**) Western Blots and densitometry for phosphorylation of Akt, as described in (**B**). Data are mean ± SEM from 5 independent experiments, with 2 biological replicates, each. * *p* < 0.05, ** *p* < 0.01, *** *p* < 0.001 vs. control, unless otherwise indicated.

Treatment of adult rat cardiomyocytes with CTRP2, CTRP7, CTRP9 and CTRP13, but not others, resulted in a significantly enhanced glucose uptake (Figure 2A). Compared to the insulin (100 nM)-mediated glucose uptake, CTRP2 was the most potent among all CTRPs (Figure 2A). Since only CTRP2, 7, 9, and 13 induced glucose uptake by cardiomyocytes, these CTRPs were further investigated for the possible molecular mechanisms. Since both AMPK and Akt signaling pathways are the major regulators of insulin-mediated glucose uptake, an activation of both pathways was investigated. As shown in Figure 2B,C, CTRP2, CTRP7, CTRP9 and CTRP13, indeed, induced phosphorylation of AMPK and Akt within 5–10 min.

**Figure 3:**
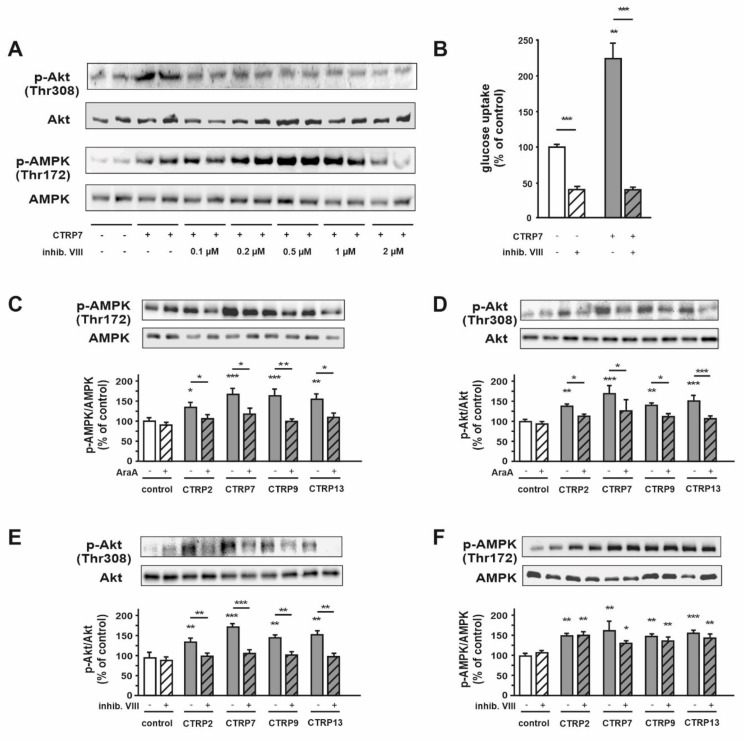
Role of AMPK and Akt on CTRP-induced effects in adult rat cardiomyocytes. (**A**) Cells were treated with CTRP7 for 10 min after preincubation with the Akt inhibitor VIII at the indicated concentrations for 30 min. Phosphorylation of Akt and AMPK were determined by Western Blotting. Total AMPK or total Akt served as loading control. Data are mean ± SEM from 5 independent experiments with 2 biological replicates, each. (**B**) Glucose uptake was measured after preincubation with the Akt inhibitor VIII (2 µM) for 30 min followed by CTRP7 for 10 min. Data are mean ± SEM of 5 independent experiments with 2 biological replicates, each. * *p* < 0.05, ** *p* < 0.01, *** *p* < 0.001 vs. untreated control, unless otherwise indicated. Phosphorylation of AMPK and Akt was investigated after preincubation with (**C**,**D**) AraA (500 µM) or (**E**,**F**) Akt inhibitor VIII (0.1 µM) for 30 min, followed by 10 min treatment with CTRP2, CTRP7, CTRP9 or CTRP13. Total AMPK or total Akt served as loading control. Data are mean ± SEM from 5 independent experiments with 2 biological replicates, each.

In order to get a further insight into the interaction between Akt and AMPK signaling, we tested increasing concentrations of the Akt inhibitor VIII (inhibitor. VIII) prior to stimulation of adult cardiomyocytes with CTRPs. Experiments with CTRP2, 7, 9 and 13 suggested that a high concentration of the inhibitor VIII (2 µM) was sufficient to completely inhibit the CTRP-induced AMPK activation, while lower concentrations (0.1 to 1 µM) resulted in an even increased AMPK phosphorylation, as shown in Figure 3A and Supplementary Figure S3. However, not only CTRP7-induced glucose uptake, but also basal glucose uptake was inhibited by 2 µM Akt inhibitor VIII (Figure 3B). Therefore, we used 0.1 µM inhibitor VIII for the next experiments. Inhibition of AMPK with AraA or inhibition of Akt with inhibitor VIII abrogated CTRP-induced AMPK and Akt phosphorylation, respectively (Figure 3C,E). In addition, phosphorylation of Akt at Thr308 was decreased in the presence of AMPK inhibitor (Figure 3D), while inhibition of Akt showed no effect on AMPK phosphorylation at Thr172 (Figure 3F), suggesting that Akt may act as a downstream target of AMPK signaling in cardiomyocytes.

**Figure 4:**
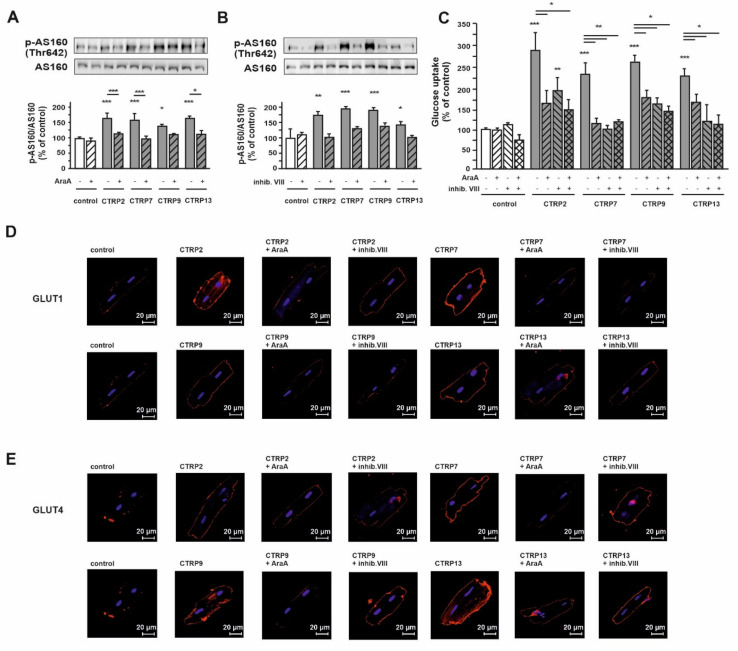
Role of AMPK and Akt inhibition on the effects of CTRP-induced glucose uptake in adult rat cardiomyocytes. AS160 (**A**,**B**) and glucose uptake (**C**) were investigated after preincubation with (**A**,**C**) AraA (500 µM) or (**B**,**C**) Akt inhibitor VIII (0.1 µM) for 30 min, followed by 10 min (**A**,**B**) or one hour (**C**) treatment with CTRP2, CTRP7, CTRP9 and/or CTRP13. Total AS160 served as loading control in A and B. Data are mean ± SEM from 5 independent experiments with 2 biological replicates, each. * *p* < 0.05, ** *p* < 0.01, *** *p* < 0.001 vs. untreated control, unless otherwise indicated. (**D**,**E**) Following AraA or VIII preincubation for 30 min and stimulation of CTRP2, CTRP7, CTRP9 and CTRP13 for 20 min, GLUT1 (**D**) and GLUT4 (**E**) were analyzed by confocal microscopy. Data are mean ± SEM from 5 independent experiments with 1 biological replicate, each.

Phosphorylation of Akt substrate of 160 kDa (AS160), which is phosphorylated on multiple sites by the Akt, induces translocation of GLUT4 to the plasma membrane [34]. CTRP2, CTRP7, CTRP9 and CTRP13 also induced an increased phosphorylation of AS160 (Figure 4A,B). Inhibition of either AMPK or Akt resulted in a significantly reduced AS160 phosphorylation (Figure 4A,B). Accordingly, pharmacological inhibition of either AMPK or Akt attenuated CTRP2, CTRP7, CTRP9 or CTRP13-induced glucose uptake (Figure 4C). Confocal microcopy showed that CTRP2 and CTRP7 treatment resulted in a strongly increased translocation of GLUT1 to the cell membrane in adult cardiomyocytes, while CTRP9 and CTRP13 treatment mediated a more moderate effect on GLUT1 translocation (Figure 4D). AraA completely blocked this GLUT1 translocation, while the Akt inhibitor VIII conveyed only a partial inhibition (Figure 4D). However, CTRP2, 7, 9 and 13 all induced GLUT4 translocation, with the weakest effect mediated by CTRP2. These CTRP effects were completely blocked by the AMPK inhibitor but not by the Akt inhibitor (Figure 4E).

### 3.3. Effect of CTRPs on Gene Expression Involved in Glucose or Fatty Acid Metabolism in Adult Rat Cardiomyocytes

**Figure 5:**
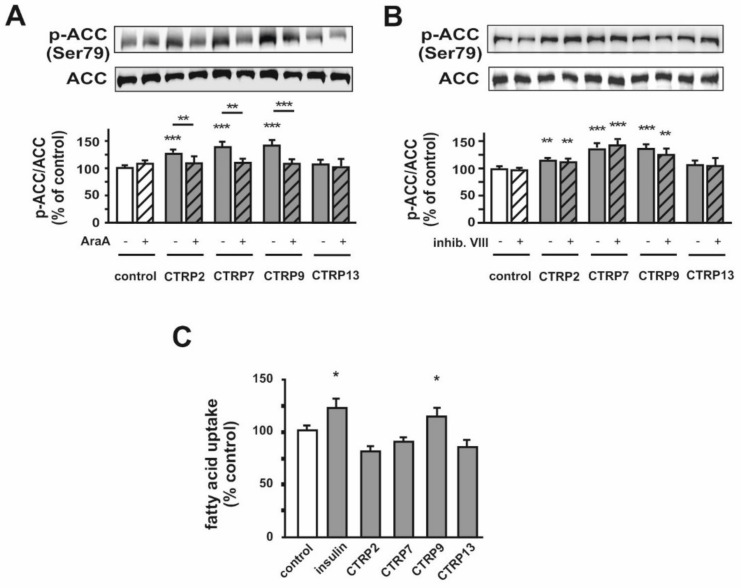
Effects of CTRPs on fatty acid metabolism in adult rat cardiomyocytes. Cells were treated with CTRP2, CTRP7, CTRP9 and CTRP13 following AraA (500 µM) (**A**) or Akt inhibitor VIII (0.1 µM) (**B**) preincubation and analyzed for the phosphorylation of ACC by Western Blotting. Total ACC served as loading control. Representative Western Blots and densitometry are shown. Data are mean ± SEM from 5 independent experiments with 2 biological replicates, each. (**C**) Fatty acid (FA) uptake was detected using the fluorescent long-chain FA analog BODIPY™ 500/510 C1, C12, following treatment with insulin, CTRP2, CTRP7, CTRP9 and CTRP13. Data are mean ± SEM from 5 independent experiments with 2 biological replicates, each. * *p* < 0.05, ** *p* < 0.01, *** *p* < 0.001 vs. untreated control, unless otherwise indicated.

Next, we examined the effect of exogenous CTRPs on the expression pattern of genes involved in glucose metabolism. Treatment with CTRP7 but not CTRP2, 9 and 13 resulted in an upregulation of GLUT1, while CTRP7 and 9 but not CTRP2 and 13 resulted in upregulation of GLUT4 expression. All the CTRPs tested caused an upregulation of hexokinase but not phosphofructokinase-1 (PFK1) (Supplementary Figure S4). However, AMPK activation has not only an effect on glucose metabolisms but also leads to an increased phosphorylation and, thus, inactivation of acetyl-CoA carboxylase (ACC) [35], the key enzyme of FA synthesis. CTRP2, CTRP7 and CTRP9 but not CTRP13 (Figure 5) induced phosphorylation of ACC within 10 min, suggesting inhibition of FA synthesis. These effects could be attenuated by inhibition of AMPK (Figure 5A), while the Akt inhibitor VIII did not abrogate the CTRPs-induced ACC phosphorylation (Figure 5B). Moreover, FA uptake could only be induced by CTRP9, while CTRP2, CTRP7 or CTRP13 (Figure 5C) did not alter FA uptake in adult cardiomyocytes. However, the CTRPs tested caused an increase in mRNA expression of ACC1, CD36, FA transport protein 1 (FATP1), very long chain acyl-CoA dehydrogenase (VLCAD), long chain acyl-CoA dehydrogenase (LCAD) or medium chain acyl-CoA dehydrogenase (MCAD) (Supplementary Figure S5). The collective upregulation of these acyl-CoA dehydrogenases suggests a stimulating impact of CTRP2, CTRP7 and CTRP9 on FA oxidation.

### 3.4. Effect of CTRPs on Glucose Uptake in H9C2 Cardiomyoblast

**Figure 6:**
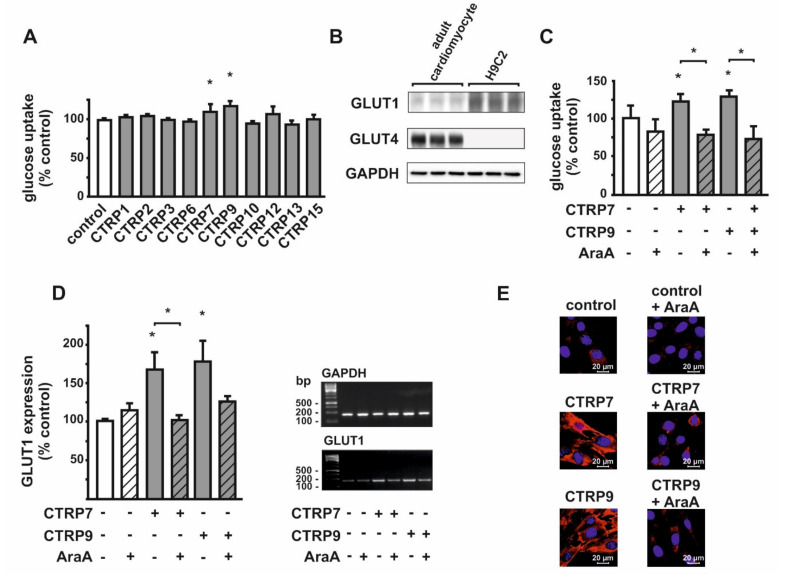
Glucose utilization and translocation of glucose transporter 1 (GLUT1) in response to CTRPs in H9C2 cardiomyoblasts. (**A**) Following glucose starving for 3 h in DMEM without glucose and serum, cells were treated with CTRPs in serum-free DMEM containing 2-[^14^C] DG for 1 h. Value was normalized to protein concentration. Data are from 5 independent experiments with 3 biological replicates, each, and are represented as mean ± SEM. * *p* < 0.05 vs. control. (**B**) Protein expression level of GLUT1 and GLUT4 in adult rat cardiomyocytes and H9C2 cells. (**C**) Effects of AMPK on basal and CTRP7- or CTRP9-induced glucose uptake in H9C2 cells. Data are mean ± SEM from 5 independent experiments with 3 biological replicates, each. * *p* < 0.05 vs. control, unless otherwise indicated. (**D**) Effects of AMPK inhibition with AraA on CTRP7- and CTRP9-induced gene expression of GLUT1. * *p* < 0.05 vs. control, unless otherwise indicated. (**E**) Microscopic images of the GLUT1 translocation in response to CTRP7 and CTRP9 treatment. The nucleus was stained with TO-PRO™-3 Iodide (642/661). GLUT1 was visualized with Cy3. Images are representatives of 3 independent experiments showing similar trends.

Unlike cardiomyocytes (Figure 2A), only CTRP7 and CTRP9 induced the glucose uptake in H9C2 cell line (Figure 6A). Therefore, the effects of CTRP7 and CTRP9 were analyzed in more detail in H9C2 cells. As shown previously by others [36], H9C2 cells expressed mainly GLUT1 (Figure 6B), unlike adult cardiomyocytes. Both CTRP7 and 9 caused an enhanced glucose uptake that was abrogated by AMPK inhibition (Figure 6C). Likewise, both CTRP7 and CTRP9 induced the GLUT1 mRNA expression, which was attenuated in the presence of AMPK inhibitor (Figure 6D). The enhanced glucose uptake was accompanied by increased GLUT1 translocation to the membrane (Figure 6E).

**Figure 7:**
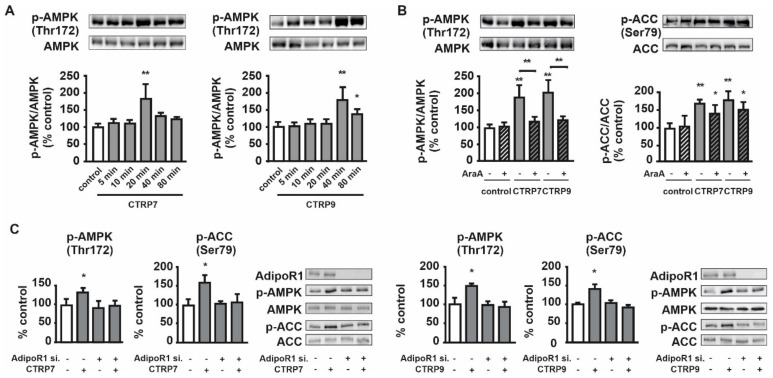
Effects of CTRP7 and CTRP9 on phosphorylation of AMPK and ACC in H9C2 cardiomyoblasts. (**A**) Western Blot analysis showing the time course of AMPK phosphorylation in response to CTRP7 or CTRP9. (**B**) Cells were incubated in the absence or presence of AMPK inhibitor AraA (500 µM) for 30 min, followed by treatment with CTRP7 for 20 min or CTRP9 for 40 min. Phosphorylation of AMPK and ACC was analyzed by Western Blot. (**C**) Western blot analysis showing phosphorylation of AMPK and ACC in mock or AdipoR1 siRNA transfected H9C2 cells, treated with CTRP7 or CTRP9, as described above. Total AMPK or total ACC served as loading control (**A**–**C**). Data are mean ± SEM from 5 independent experiments with 3 biological replicates, each. * *p* < 0.05, ** *p* < 0.01 vs. control, unless otherwise indicated.

Comparable to the data obtained in cardiomyocytes, CTRP7 induced a strong phosphorylation of AMPK within 20 min, while phosphorylation of AMPK by CTRP9 occurred within 5 min, reaching a maximum after 40 min (Figure 7A). Following pretreatment with AraA, AMPK activation by CTRP7 and CTRP9 was totally abolished, while phosphorylation of the AMPK downstream target ACC by CTRP7 and CTRP9 remained largely unaffected (Figure 7B). This suggests that the phosphorylation on ACC by CTRP7 and CTRP9 is not mainly dependent on AMPK activation. Treatment of H9C2 cells with CTRP7 or CTRP9 resulted in a mild activation of Akt (Supplementary Figure S6). Since there are structural similarities between adiponectin and the CTRPs, and adiponectin has been shown to mediate its metabolic effects mostly via AdipoR1 and AdipoR2 [6,27], the CTRPs were also suggested to utilize the adiponectin receptors [28]. Similar to the data obtained in skeletal muscle, adult cardiomyocytes and H9C2 cells express mainly AdipoR1 but little AdipoR2, which shows highest expression in liver [27,29]. To evaluate the role of AdipoR1 in mediating the effects of CTRP7 and CTRP9, H9C2 cardiomyoblasts were transfected with AdipoR1 siRNA (Figure 7C). This knockdown abolished the CTRP7- and CTRP9-mediated phosphorylation of AMPK and ACC, suggesting a major role of AdipoR1 in mediating these effects (Figure 7C). Knockdown of AdipoR2 in H9C2 cardiomyoblasts did not alter the CTRP effects on AMPK signaling (not shown).

**Figure 8:**
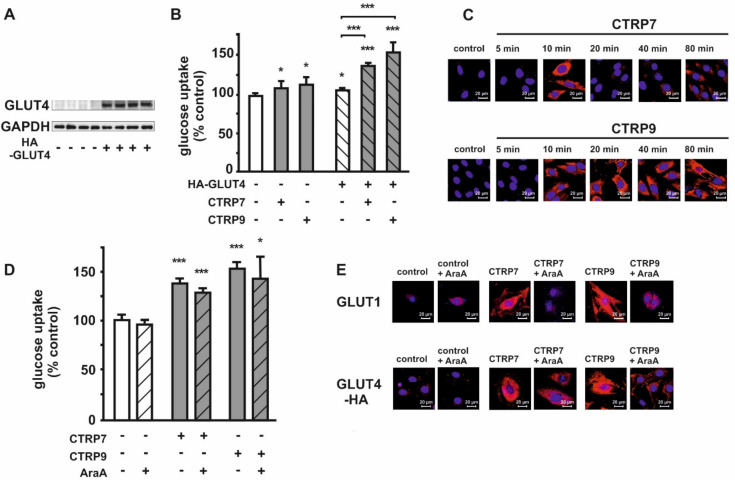
Effects of CTRP7 and CTRP9 on glucose uptake in GLUT4-overexpressing H9C2 cardiomyoblasts. (**A**) Western Blot identification of GLUT4 expression following GLUT4 overexpression compared to basal GLUT4 expression in H9C2 cells. GAPDH served as loading control. (**B**) Following glucose starving for 3 h in DMEM without glucose and serum, cells were treated with CTRPs in serum-free DMEM containing 2-[^14^C] DG for 1 h. Value was normalized to protein concentration. Data are from 5 independent experiments with 3 biological replicates, each, and are represented as mean ± SEM. * *p* < 0.05, *** *p* < 0.001 vs. control, unless otherwise indicated. (**C**) Time course of GLUT4 translocation in response to CTRP7 or CTRP9, as detected with an anti-HA antibody. The nucleus was stained with TO-PRO™-3 Iodide. (**D**) Effects of AMPK inhibition with AraA (500 µM) on CTRP7 and CTRP9 induced glucose uptake in H9C2 cells with GLUT4 overexpression. Data are presented as mean ± SEM of 5 independent experiments. * *p* < 0.05, *** *p* < 0.001 vs. control, unless otherwise indicated. (**E**) Effects of AMPK inhibition with AraA (500 µM) on CTRP7- and CTRP9-induced GLUT1 and GLUT4 translocation. Images are representatives of 3 independent experiments showing similar trends.

In order to evaluate the impact of the CTRPs on GLUT4 translocation and GLUT4 mediated glucose uptake in H9C2 cardiomyoblasts, the cells were transfected with pQBI-HA-GLUT4, resulting in the overexpression of a HA-tagged GLUT4 with a HA epitope in an exofacial loop of GLUT4 (Figure 8A). H9C2 cells with GLUT4 overexpression demonstrated a higher basal glucose uptake than native H9C2 cells, as well as a significantly stronger increase in glucose uptake following treatment with CTRP7 and CTRP9 (Figure 8B). In a further step, the translocation of GLUT4 was detected with an anti-HA antibody, showing that CTRP7 led to a strong signal of HA-GLUT4 only after 10 min, while CTRP9 induced HA-GLUT4 translocation after 20 min and reached its maximal level after 80 min (Figure 8C). Following HA-GLUT4 overexpression, the inhibitory effect of AraA on glucose uptake, as observed in native H9C2 cells (Figure 6C), was almost completely abolished (Figure 8D). Similarly, confocal microcopy showed that inhibition of AMPK with AraA resulted in a significant reduction of CTRP7- and CTRP9-mediated translocation of GLUT1, while GLUT4 translocation was only partially inhibited by AraA in H9C2 cardiomyoblasts with HA-GLUT4 overexpression (Figure 8E). This suggests that the high level of GLUT4 that we achieved by overexpression resulted in an escape from AMPK inhibition (Figure 8D,E), compared to native H9C2 cells (Figure 6C).

The metabolic changes observed in H9C2 cells in response to CTRP7 and CTRP9 were associated with an increased viability, compared to control cells, as deduced from measurements of their overall metabolic activity (Supplementary Figure S7). In addition, ATP content was significantly increased, but ROS production was reduced following 24 h of CTRP treatment (Supplementary Figure S7). The latter effect of CTRP9 has previously been reported by our group and by others [29,37]. In total, this suggested that the stimulation of cardiomyocyte metabolism by CTRPs does not result in detrimental cellular effects.

## 4. Discussion

The main findings of present study are (1) CTRP2, 7, 9, and 13 induce glucose uptake in rat cardiomyocytes via AMPK/Akt-mediated GLUT4 translocation to membrane; (2) CTRP2, 7, 9, and 13 modulate cardiomyocyte FA metabolism; and (3) these CTRPs effects are mediated via AdipoR1.

Consistent with previous studies from others [9], we report here an almost ubiquitous distribution of all CTRPs. However, despite this wide distribution, the plasma concentration of all CTRPs is known to be far lower than adiponectin, although their local tissue concentration may be much higher than adiponectin [26], suggesting that CTRPs may be acting locally in a paracrine and/or autocrine fashion [5]. Adiponectin has been shown to induce glucose uptake in cardiomyocytes [38], while CTRP-mediated effects on glucose uptake/metabolism in cardiomyocytes are largely unknown. In cardiomyocytes, glucose uptake occurs mainly via GLUT1 and GLUT4 [39]. While GLUT1 is regarded as the basal glucose transporter, GLUT4 is responsible for the increase in glucose uptake upon stimulation. Under basal conditions, GLUT4 is stored in transport vesicles and is actively transferred to membrane in response to insulin or increased workload [40]. Although GLUT1 is considered to be constitutively located at the plasma membrane, the presence of a cytoplasmic pool of GLUT1 has also been reported in cardiomyocytes [41]. A small stimulating impact of insulin or ischemia on GLUT1 translocation exists [41], but it is likely that GLUT4-mediated uptake represents the major mechanism under these conditions. Interestingly, the increased glucose uptake by hypertrophied hearts is associated with an increased expression of GLUT1 but not GLUT4 [42].

In the present study, we demonstrate that CTRP2, 7, 9 and 13 induce a translocation of both GLUT1 and GLUT4 in adult rat cardiomyocytes, suggesting both may be responsible for the CTRP-mediated enhanced glucose uptake. In contrast to adult cardiomyocytes, H9C2 cardiomyoblasts mainly express GLUT1 and largely lack GLUT4, comparable to developing cardiomyocytes [41]. In these cells, CTRP2 and CTRP13 are unable to induce glucose uptake, suggesting that GLUT4 but not GLUT1 may be the main target of these CTRPs, as lack of GLUT4 results in loss of their effects in H9C2 cells. CTRP7 and CTRP9, however, are able to weakly induce glucose uptake (Figure 6A) and GLUT1 translocation (Figure 6E) in H9C2 cells. Interestingly, ectopic expression of GLUT4 potentiates the CTRP7- and CTRP9-mediated glucose uptake in these cells (Figure 8B). These data suggest that CTRP7 and CTRP9 induce glucose uptake in cardiomyocytes mainly via GLUT4 translocation and partly via GLUT1. Unlike the strong effects observed on glucose uptake, only minor stimulating effects on FA uptake were induced by CTRP9 (Figure 5C). However, the effects of CTRP2, 7 and 9 on ACC phosphorylation suggest a stimulatory effect of these CTRPs on FA oxidation, although a direct measurement was not performed. Similarly, others have described a stimulation of FA uptake only for few CTRPs, while various CTRPs appear to stimulate FA oxidation [5,15,18,19,30]. The mechanisms leading to the observed upregulation of genes involved in FA metabolism, such as the acyl-CoA dehydrogenases (Supplementary Figure S5), which may involve the activation of Peroxisome proliferator-activated receptor alpha (PPARalpha) or peroxisome proliferator-activated receptor gamma coactivator-1 alpha or beta [43,44], will require further investigations.

Cardiac insulin-induced GLUT4 and GLUT1 translocation is mediated via activation of phosphatidylinositol 3-kinase (PI3K)/protein kinase B (PKB/Akt) pathway [45]. Activation of Akt is regulated by phosphorylation at two different sites by different kinases. Phosphorylation at T308 by phosphoinositide-dependent protein kinase 1 (PDK1) is essential for its active kinase conformation and responsible for GLUT4 translocation [46]. Additional phosphorylation at S473 by mTORC2 enhances its activity and contributes to substrate specificity [46]. Activated Akt phosphorylates and inhibits its substrate of 160 kDa (AS160) and relieves the inhibition of Rab-GTPases, thus facilitating the translocation of GLUT-containing vesicles to the membrane [34,47]. AS160 is also phosphorylated and inhibited by AMPK, which offers an alternative, insulin-independent mechanism of GLUT translocation to membrane [47,48]. In the present study, we investigated the activation of AMPK and Akt signaling pathways as potential mechanisms of CTRP-mediated GLUT1 and GLUT4 translocation and enhanced glucose uptake in cardiomyocytes. We demonstrate an induction of AMPK phosphorylation at T172 and Akt at T308 by CTRPs. Pharmacological inhibition of AMPK totally abrogates GLUT1 and GLUT4 membrane translocation, as well as glucose uptake induced by CTRPs, confirming their role in cardiomyocytes, while inhibition of Akt only partially abolishes CTRP-induced GLUT1 translocation. CTRP-induced Akt phosphorylation is abrogated by pharmacological inhibition of AMPK but not vice-versa, suggesting that AMPK signaling lies upstream to Akt activation. Similarly, it has been shown that AMPK activation by the antidiabetic drug metformin improves cardiac insulin sensitivity by enhancing PKB/Akt signaling [49]. The insulin sensitizing effects of AMPK on PKB/Akt were suggested to be due to its counteracting effect on the insulin-negative feedback loop involving the mTOR/p70 ribosomal S6 protein kinase pathway [47]. However, the increase of glucose uptake appears to be independent of this [47] and probably involves additional mechanisms, such as direct phosphorylation of AS160 by AMPK or activation of the Rac1–PAK–cofillin pathway [47,48,50].

Adiponectin effects are mediated via its two receptors AdipoR1 and AdipoR2 [27]. Other studies suggested that some actions of adiponectin may be mediated through additional receptors, including PAQR3, which is a member of the same receptor family as AdipoR1/2 [51]. Additionally, two AdipoR binding proteins, calreticulin and T-cadherin, have been reported to modify the AdipoR affinity, the latter being essential for the cardioprotective actions of adiponectin [29,52,53]. AdipoR1 is more prominent in muscle tissue and seems to be linked to AMPK activation, while AdipoR2 is more prominent in liver cells and is linked to PPARalpha activation [27]. CTRP2, 7, 9 and 13 show highest homology to adiponectin [9] and may, therefore, share its pharmacological and functional properties. Indeed, CTRP2, 7, 9 and 13, but no other CTRP, enhance glucose uptake in adult rat cardiomyocytes in the present study. In order to investigate which of the two adiponectin receptors is responsible for the observed effects in cardiomyocytes, we used H9C2 myoblasts, as they are easily genetically manipulated. Knockdown of AdipoR1 but not AdipoR2 completely abolishes the CTRP7- and CTRP9-induced AMPK and ACC phosphorylation in H9C2 cells. These data suggest that CTRP 2-, 7-, 9- and 13- induced glucose uptake in cardiac muscle cells occurs mainly via AdipoR1. The differences observed between the CTRPs in mediating effects on glucose metabolism in cardiomyocytes may be related to variations in receptor/co-receptor affinity, formation of distinct hetero- or homodimers between CTRPs, varying posttranslational modifications [5,6,54], but these were not investigated in detail in the present study.

Study limitations: Adult cardiomyocytes are hard to transfect, and long-term culturing compromises their viability and results in dedifferentiation. Therefore, we used H9C2 cells instead of primary cardiomyocytes for receptor investigation. These cells have the ability to differentiate toward a cardiac phenotype and show energy metabolism patterns similar to primary cardiomyocyte [55,56]. However, a clear disadvantage of H9C2 cardiomyoblasts is that they are characterized by high GLUT1 expression and poor insulin responsiveness, rendering them more comparable to embryonic or neonatal cardiomyocytes than to adult cardiomyocytes [40]. Therefore, future experiments need to find better strategies to genetically manipulate the receptors. In addition, the utilization of recently described pharmacological AdipoR antagonists [57] may help to define the role of adiponectin receptors in mediating the metabolic CTRP effects in cardiomyocytes.

## 5. Conclusions

We show that CTRP2, 7, 9 and 13 induce glucose uptake via AdipoR1-dependent activation of AMPK/Akt signaling and GLUT1 and GLUT4 translocation in adult rat cardiomyocytes. In metabolic diseases like diabetes mellitus, insulin signaling is disturbed, leading to an impaired glucose homeostasis. Targeting the AMPK signaling pathway to promote glucose utilization via CTRP may offer a therapeutic target to restore glucose homeostasis by acting on glucose uptake independently of the PI3K/PKB/Akt pathway and, thus, reduce insulin resistance.

## Data Availability

The data presented in this study are available on request from the corresponding author.

## Supplementary Materials

The following are available online at https://www.mdpi.com/article/10.3390/cells10040905/s1, Figure S1: Overexpression of CTRPs in *E. coli* BL21-AI, Figure S2: Purification of CTRPs from *E. coli* BL21-AI, Figure S3: Role of AMPK and Akt on CTRP-induced effects in adult rat cardiomyocytes, Figure S4: Effects of CTRPs on the expression of genes involved in glucose metabolism, Figure S5: Effects of CTRPs on the expression of genes involved in fatty acid metabolism, Figure S6: Effects of CTRP7 and CTRP9 on phosphorylation of Akt in H9C2 cardiomyoblasts, Figure S7: Impact of CTRPs on cell viability, ATP content and ROS production. Table S1: Primer sequences.
