# Supplementary material for: Comparative Analysis of CTRP-Mediated Effects on Cardiomyocyte Glucose Metabolism: Cross Talk between AMPK and Akt Signaling Pathway
Source: Cells. 2021 Apr 14;10(4):905. doi: 10.3390/cells10040905 (PMC8070942; doi:10.3390/cells10040905)
Supplement: Supplementary file 1 [file cells-10-00905-s001.pdf]

Comparative analysis of CTRP-mediated effects on cardiomyocyte glucose metabolism: Cross-talk between AMPK and Akt signaling pathway

Ling Li[1,*], Muhammad Aslam[2,*], Benedikt H. Siegler[1], Bernd Niemann[3], Susanne Rohrbach[1]

[1]Institute of Physiology, [2]Department of Cardiology and Angiology and [3]Department of Cardiac and Vascular Surgery, Justus Liebig University Giessen

* Both authors contributed equally.

Corresponding author:
Dr. Ling Li
Institute for Physiology
Justus Liebig University Giessen
Aulweg 129
35392 Giessen
Germany
Ling.Li@physiologie.med.uni-giessen.de
Phone: 0049-641-9947342
Fax: 0049-641-9947269

Supplement

Table 1: Primer sequences

	**GenBank accession #**	**Forward Primer**	**Reverse Primer**
CTRP1	NM_001007675	CTT CCA GCC TGT GGT CTT CG	GCA GGT AGG TCT CCT TCT GGT
CTRP2	NM_001191918	GGA CCT AAG GGC AAG AAA GG	GTA GTG GCC TCC CTC ATT CA
CTRP3	NM_001134436.1	TGA TGA AGC ATG AGG ACG TG	TGC ATG GTT GCT GGA TGT AT
CTRP4	NM_001107745.1	TGA TTT ACG ACG ATG GCG CT	AGC AGT GAG GTC AGG GTA CA
CTRP5	NM_001012123	TCT CAG GGG GTG CGA TGG T	AGC GAA GAC TGG GGA GCT GT
CTRP6	NM_001034932.1	GCA TTA CTC GGC CTT CTC TG	GTG CAC ATT GAG GCT GAA GA
CTRP7	NM_001107221	CCC ATA GGA CCA GAG GGT GA	TGA GCA CGA TGC TTC CAC AT
CTRP9	NM_001191891	GGT GGC TTC TAC TGG TTA TGG A	CTC TCC ACG AAT TCC ATC CTT
CTRP10	NM_001105949.1	GTG ACC CGT ACA CCG TCT CT	TGG TCC CTG GAT AAA CGG AGG
CTRP12	NM_001108000	CCT GTC CTT GGG CCG ATT TA	CAG GGA CGT ATG ACG GTG AC
CTRP13	NM_001109403	AGA GCC CGG ACC ACC GGG	GCG ATC TTG GGC ACC GTG CT
CTRP15	NM_001377080.1	CGG AGT CTA CTA CCT GCC CG	TGG TGG TCC CTT TCT TGG CT
ACC1	NM_022193.1	AGT GGC AGT GGT CTT CGA GT	TCC ATC ATC CAC ATC CTT CA

FATP1	U89529.1	GTG GTG CAC AGC AGG TAC TA	TTG CGC AGT ACC ACC GTT A
CD36	NM_031561.2	TGC AGG TCA ACA TAC TGG TCA A	CCC GGT CAC TTG GTT TCT GA
LCAD	NM_012819.1	TCA TGC AAG AGC TCC CAC AG	GCA GCT GTC CAC AAA AGC TC
VLCAD	NM_012891.2	CCT CTG CCC AGC GAC TTT AT	CCT CTG CCC AGC GAC TTT AT
MCAD	NM_016986.2	GGT CTT GGC CTG GGA ACT TT	AGT AGG CAC ACA TCA TCG GC
GLUT1	NM_138827	TGT GTT CTA CTA CTC AAC GAG CA	CAA TGA GAT GCA GGG TCC GA
GLUT4	NM_012751.1	GGC TGT AGC TGG TTT CTC CA	AAA TGT CCG GCC TCT GGT TT
hexokinase	NM_012735.2	TCG CAT ATG ATC GCC TGC TT	AGC TCC TAG CCC TTT CTC CA
PFK1	NM_080477	GTG GCA CTG GAA GAT GTG AA	AAT GGC AGC AAT GAC ATC AG
BNP	NM_031545	GAT CTC CAG AAG GTG CTG GG	TGT AGG GCC TTG GTC CT
eNOS	NM_021838.2	CTG CGC TGG TAT GCC CTC C	CCG CTC TGT AAC TTC CTT G
fibronectin	NM_019143.2	CCT GGA ACT TCT ACC AGT GCG ACT CT	CGT AGG CTG GTT CAG GCC TTC G
18S rRNA	NR_046237	TGG AGC GAT TTG TCT GGT TA	ACG CCA CTT GTC CCT CTA AG
GAPDH	NM_017008.4	CAT CAC CAT CTT CCA GGA GGG	GGT TCA CAC CCA TGA CGA ACA
HPRT-1	NM_012583.2	CGA GCC GAC CGG TTC TGT CAT G	GAG GGC CAC AAT GTG ATG GCC TC

Supplementary Figures

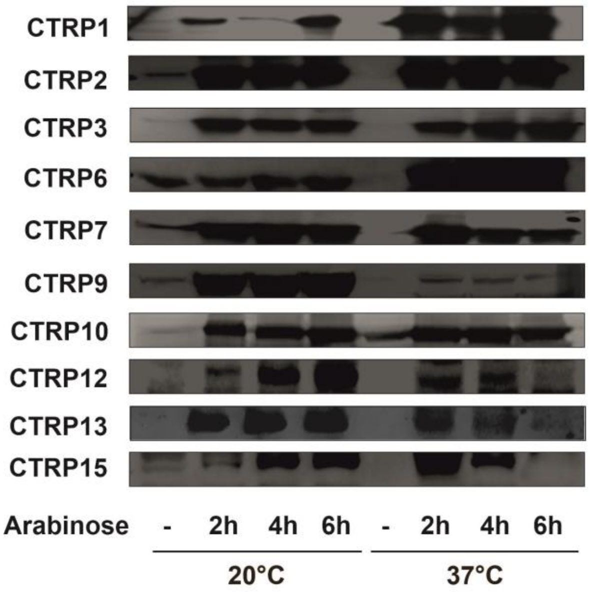

Supplementary Figure 1: Overexpression of CTRPs in *E.coli* BL21-AI. Bacterial protein expression was induced with arabinose at 20 ℃ and 37 ℃ for 2, 4, and 6 hours, followed by homogenization by sonication. Expression of CTRPs was analyzed in these lysates by Western Blotting using antibodies directed against His or MBP.

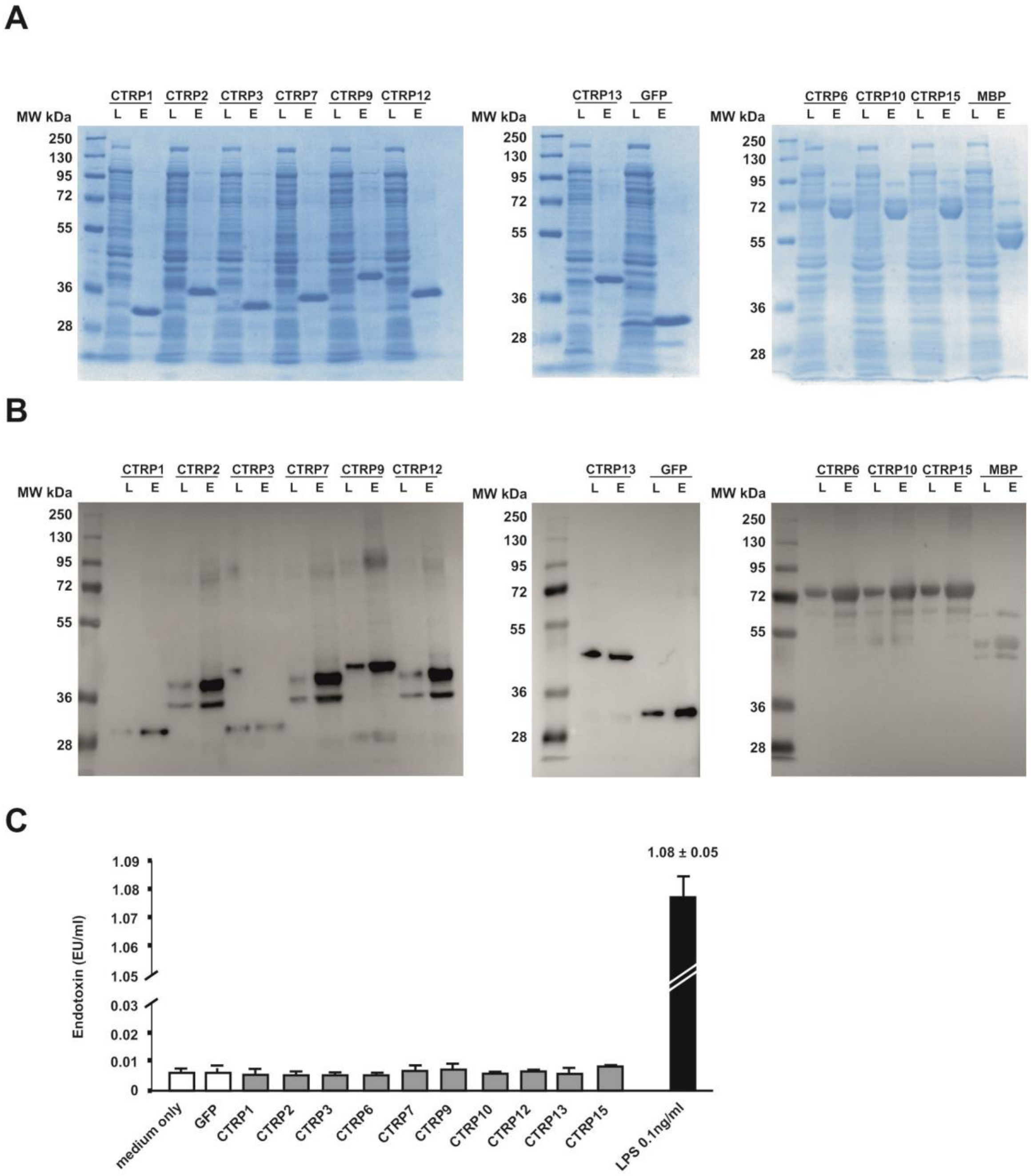

Supplementary Figure 2: Purification of CTRPs from *E.coli* BL21-AI. Overexpressed CTRP1, CTRP2, CTRP3, CTRP7, CTRP9, CTRP12, CTRP13 and GFP were purified with nickel-affinity columns. CTRP6, CTRP10, CTRP15 and MBP were purified with amylose resin. Lysate (L) and eluates (E) were analyzed in Coomassie Blue-stained SDS-PAGE gels (**A**) and by Western Blot using an anti-His antibody for CTRP1, 2, 3, 7, 9, 12, 13 and GFP or an anti-MBP antibody for CTRP6, 10, 15 and MBP. (**B**). Following removal of potential endotoxin contaminants, the absence of endotoxin was verified (**C**).

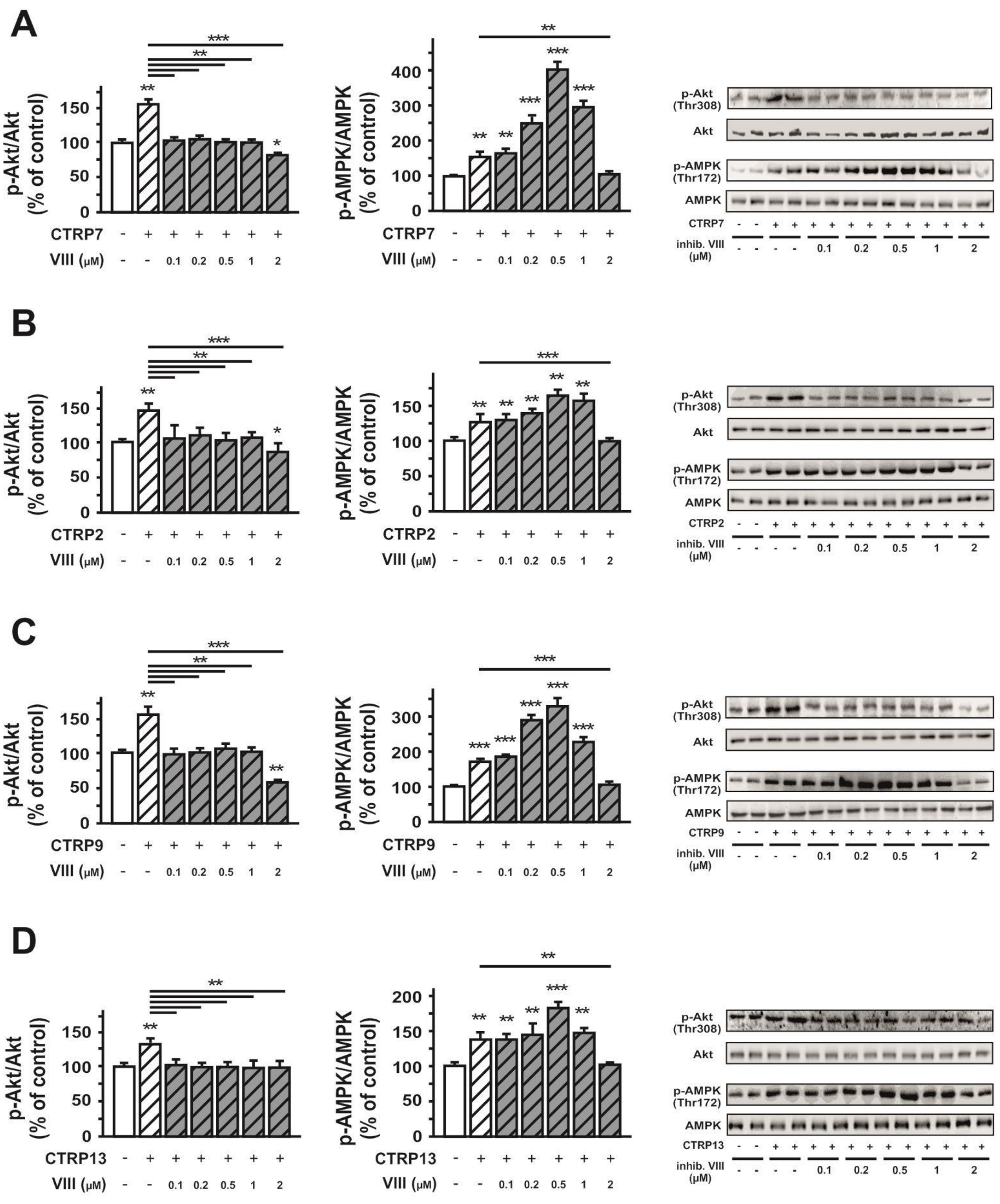

Supplementary Figure 3: Role of AMPK and Akt on CTRP-induced effects in adult rat cardiomyocytes. Cells were treated with CTRP7 (**A**), CTRP2 (**B**), CTRP9 (**C**) or CTRP13 (**D**) for 10 minutes after preincubation with the Akt inhibitor VIII at the indicated concentrations for 30 minutes. Phosphorylation of Akt and AMPK was determined by Western Blotting. Total AMPK or total Akt served as loading control. Representative Western blots and the according densitometry are shown. Data are mean ± SEM from 5 independent experiments with 2 biological replicates each. *p<0.05, **p<0.01, ***p<0.001 vs. untreated control unless otherwise indicated.

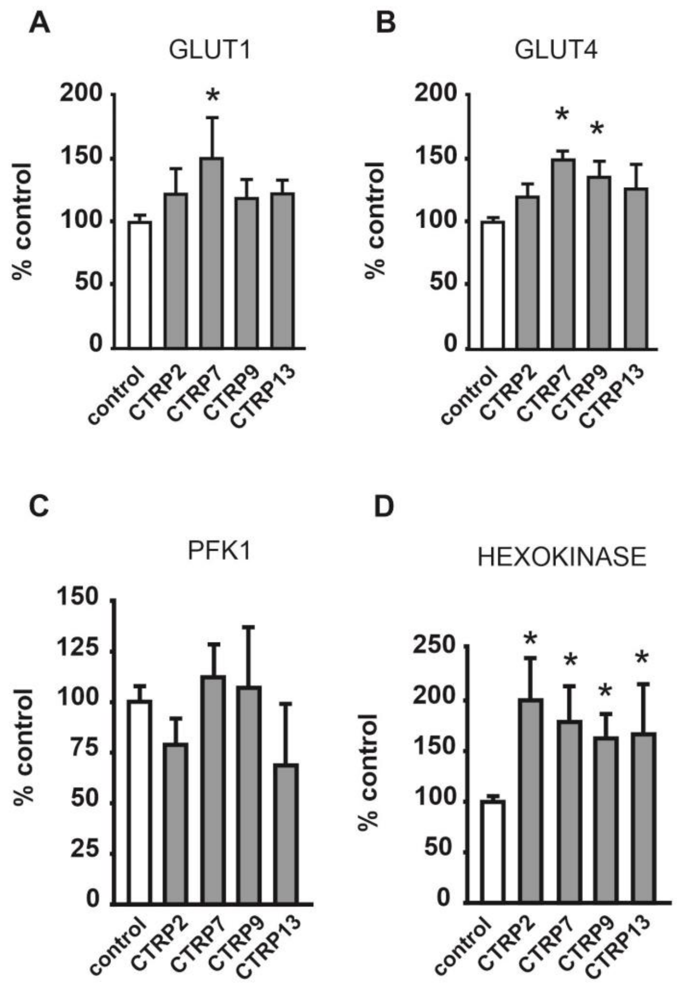

Supplementary Figure 4: Effects of CTRPs on the expression of genes involved in glucose metabolism. Adult rat cardiomyocyte were treated with CTRPs for 6 hours. mRNA levels of genes involved in glucose uptake and glycolysis were quantified by Real-time PCR. All data were normalized to 18S rRNA, GAPDH and HPRT-1 in each sample. Results are expressed as mean ± SEM of 5 independent experiments with 3 biological replicates per group. *$p<0.05$ vs. control.

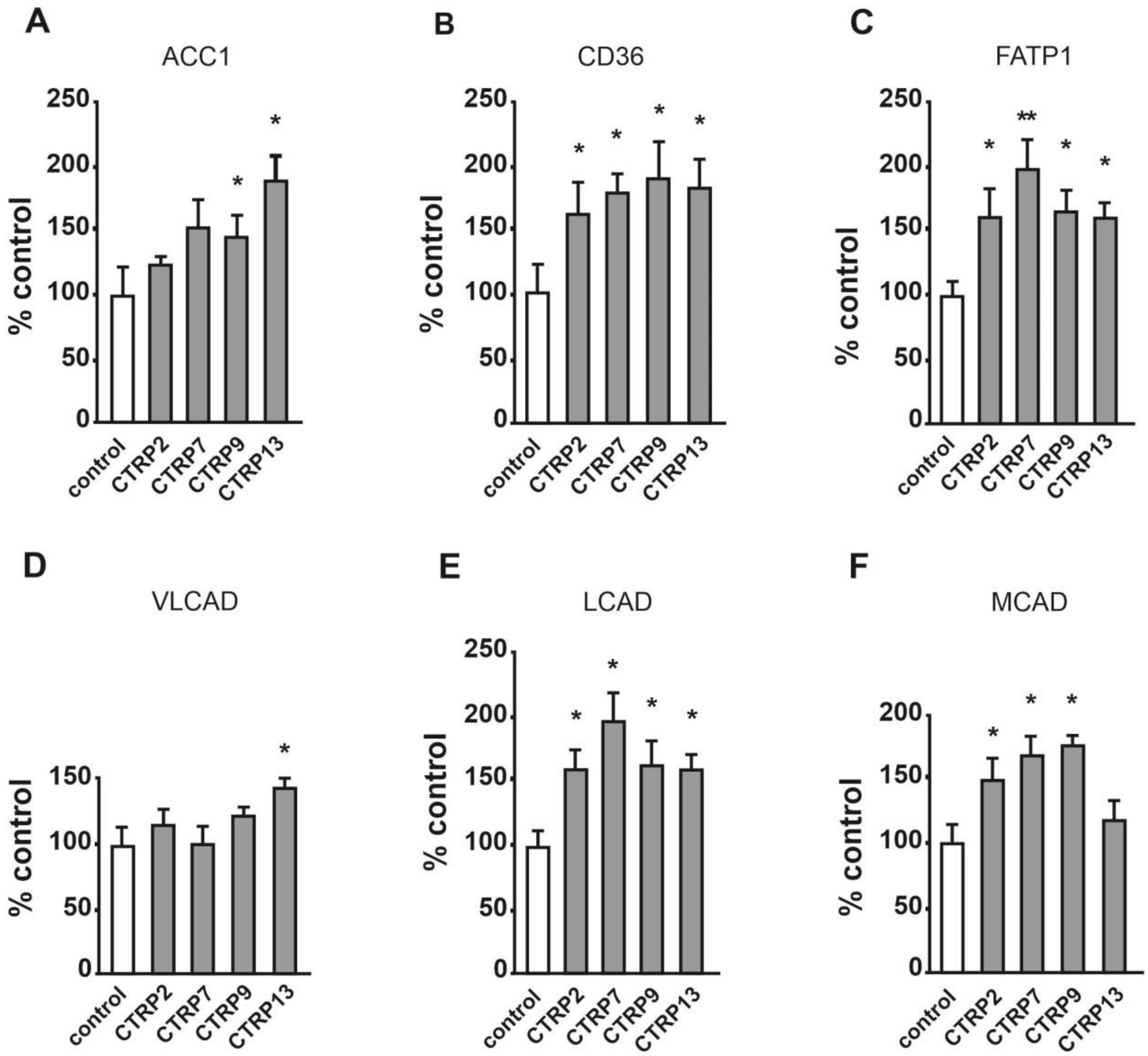

Supplementary Figure 5: Effects of CTRPs on the expression of genes involved in fatty acid metabolism. Adult rat cardiomyocyte were treated with CTRPs for 6 hours. mRNA levels of genes involved in fatty acid metabolism were quantified by Real-time PCR. All data were normalized to 18S rRNA, GAPDH and HPRT-1 in each sample. Results are expressed as mean ± SEM of 5 independent experiments with 3 biological replicates per group. *$p<0.05$, **$p<0.01$ vs. control.

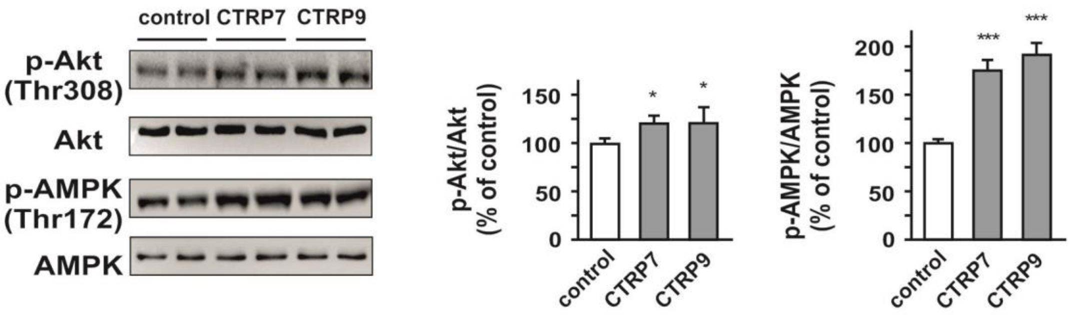

Supplementary Figure 6: Effects of CTRP7 and CTRP9 on phosphorylation of Akt in H9C2 cardiomyoblasts. Cells were treated with CTRP7 and 9 (4 µg/ml) for 30 minutes. Phosphorylation of Akt and AMPK was determined by Western Blotting. Total AMPK or total Akt served as loading control. Representative Western blots and the according densitometry are shown. Data are from 5 independent experiments with 2 biological replicates each. *$p<0.05$, ***$p<0.001$ vs. control.

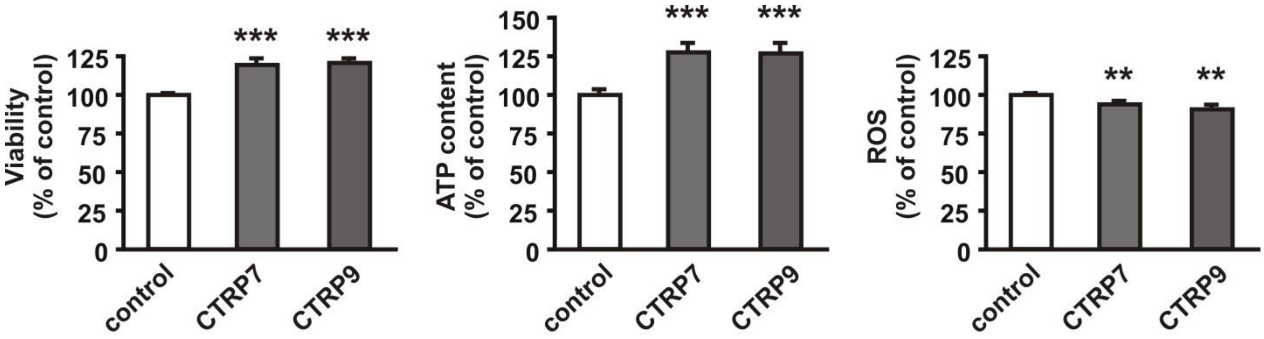

Supplementary Figure 7: Impact of CTRPs on cell viability, ATP content and ROS production. H9C2 cells were grown on 96-wells and treated with CTRP7 or CTRP9 (4 µg/ml) for 24 hours. Cell viabilty was measured as an indicator of their metabolic capacity due to their ability to reduce resazurin into resorufin. ATP concentration was determined by phosphorylating glycerol, resulting in a fluorometric product. For the measurement of cellular ROS production, cells were loaded with DCFDA. Results are expressed as mean ± SEM of 4 independent experiments with 16 biological replicates each. $^{**}p<0.01$, $^{***}p<0.001$ vs. control.
